# Measurement of plantar pressure data in children with clubfoot

**DOI:** 10.1186/1757-1146-7-S1-A79

**Published:** 2014-04-08

**Authors:** Julie Stebbins, Louise Way, Claudia Giacomozzi

**Affiliations:** 1 Oxford Gait Laboratory, Oxford University Hospitals NHS Trust, Oxford, Oxon, OX3 7HE, UK; 2 Department of Technology and Health, Istituto Superiore di Sanità, Rome, Italy

## Background

It is becoming increasingly common for pedobarography to be used to aid treatment planning. While there is some consensus on use of this data in specific populations (for example diabetes in adults) there is little information on how it should be interpreted in those with paediatric orthopaedic conditions, such as clubfoot. This is due in part to a scarcity in the literature of “normal” reference data for children, as well as a lack of consensus on the clinical interpretation of deviations from normal data. The aim of this study was to determine if the plantar pressure distribution in children with clubfeet can reliably be distinguished from an age-matched, typically developing population.

## Patients/materials and methods

**Figure 1:**
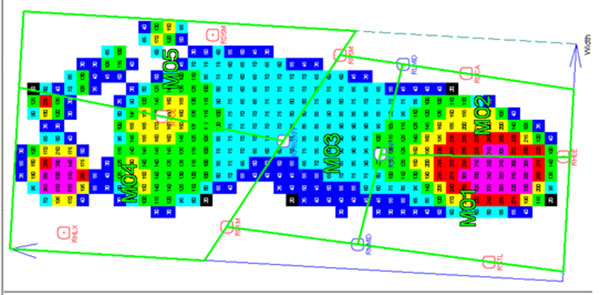
CF footprint masked based on marker co-ordinates

73 typically developing (TD) children (age 6-16 years) with no known pathology and 10 children with treated clubfoot (CF) (age 5-16 years) participated. Plantar pressure data (emed-m, novel, Germany) were obtained while walking at self-selected speed. Synchronous trajectory data were collected from reflective markers placed on the feet according to the Oxford Foot Model [1] using a 12 camera system (Vicon, Oxford, UK). Pressure images were masked into 5 areas using projected marker co-ordinates (Figure 1) [2]. The TD children were grouped into 6 age bands. Data from each CF subject was compared to age-matched data from the TD population.

## Results

**Figure 2:**
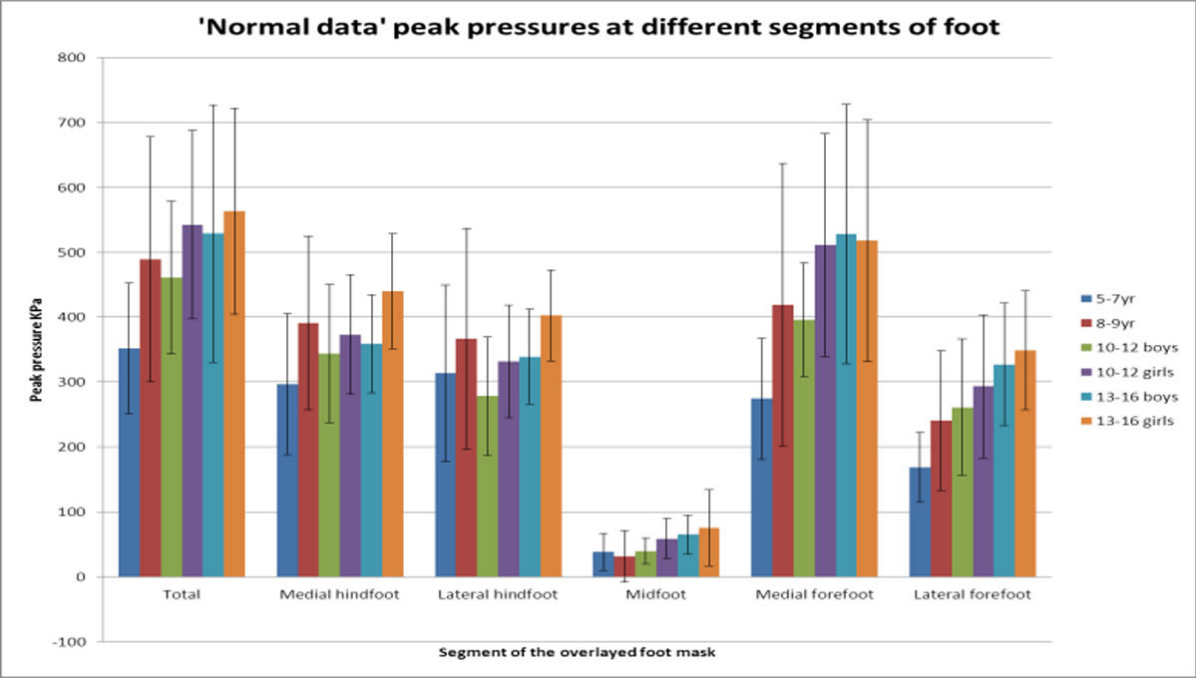
Peak pressure in each sub-area across different ages

Differences were found across the age groups in the TD population (Figure 2) with a progressive increase in peak pressure with age in most areas. 9/10 CF subjects demonstrated significant differences to the TD population in at least one sub-area of the foot. The most frequent differences were found in the mid-foot region, with 11/20 feet having increased pressure in this region. There was generally a reduction in pressure in the hindfoot region, and a mixed response at the forefoot.

## Conclusion

Substantial variation in pressure distribution was found in the TD population. Despite this, almost all CF subjects demonstrated significant differences to age-matched TD data. This suggests that pedobarography provides adequate sensitivity for assessing this population. “Normal” reference data may be used as a comparison (similar to other gait data) but care should be taken that this is appropriately age-matched. Markers placed on the foot were used to automatically mask the footprint for this study, allowing accuracy of masking to be maintained, even in the presence of abnormal foot shapes. This needs to be taken into consideration when assessing data from a clubfoot population.
